# Four decades of functional community change reveals gradual trends and low interlinkage across trophic groups in a large marine ecosystem

**DOI:** 10.1111/gcb.14552

**Published:** 2019-02-20

**Authors:** Anna Törnroos, Laurene Pecuchet, Jens Olsson, Anna Gårdmark, Mats Blomqvist, Martin Lindegren, Erik Bonsdorff

**Affiliations:** ^1^ Environmental and Marine Biology Åbo Akademi University Turku Finland; ^2^ Centre for Ocean Life, DTU‐Aqua Kngs. Lyngby Denmark; ^3^ Department of Aquatic Resources Swedish University of Agricultural Sciences Öregrund Sweden; ^4^ Hafok AB Stenhamra Sweden

**Keywords:** Baltic Sea, coastal ecosystem, community dynamics, fish, functional diversity, functional turnover, multifunctionality, temporal change, trait‐based approach, zoobenthos

## Abstract

The rate at which biological diversity is altered on both land and in the sea, makes temporal community development a critical and fundamental part of understanding global change. With advancements in trait‐based approaches, the focus on the impact of temporal change has shifted towards its potential effects on the functioning of the ecosystems. Our mechanistic understanding of and ability to predict community change is still impeded by the lack of knowledge in long‐term functional dynamics that span several trophic levels. To address this, we assessed species richness and multiple dimensions of functional diversity and dynamics of two interacting key organism groups in the marine food web: fish and zoobenthos. We utilized unique time series‐data spanning four decades, from three environmentally distinct coastal areas in the Baltic Sea, and assembled trait information on six traits per organism group covering aspects of feeding, living habit, reproduction and life history. We identified gradual long‐term trends, rather than abrupt changes in functional diversity (trait richness, evenness, dispersion) trait turnover, and overall multi‐trait community composition. The linkage between fish and zoobenthic functional community change, in terms of correlation in long‐term trends, was weak, with timing of changes being area and trophic group specific. Developments of fish and zoobenthos traits, particularly size (increase in small size for both groups) and feeding habits (e.g. increase in generalist feeding for fish and scavenging or predation for zoobenthos), suggest changes in trophic pathways. We summarize our findings by highlighting three key aspects for understanding functional change across trophic groups: (a) decoupling of species from trait richness, (b) decoupling of richness from density and (c) determining of turnover and multi‐trait dynamics. We therefore argue for quantifying change in multiple functional measures to help assessments of biodiversity change move beyond taxonomy and single trophic groups.

## Graphical abstract

In this study, we identified gradual long‐term trends in functional diversity, trait turnover, and overall multi‐trait community composition spanning a period of 40 years and two key trophic groups: fish and zoobenthos, in three coastal marine areas. The study highlights the need for multiple measures and cross‐trophic level assessments to understand empirical functional (trait) change and serves as a baseline for functional change in the Baltic Sea region and other coastal and estuarine ecosystems worldwide. The findings contribute to the general understanding of biodiversity change and can be useful for developing predictions and models of community change.

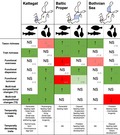

## 1 INTRODUCTION

Ecosystems worldwide are exposed to a range of natural and human‐induced pressures, including climate change, overexploitation and habitat alteration (Cardinale et al., 2012; Halpern et al., 2015; Hooper et al., 2005). These pressures alter biological diversity through regional species extinctions and invasions, as well as dominance and density (abundance and/or biomass) shifts within communities, highlighting the importance of temporal community adaptation as a fundamental part of global change (Hooper et al., 2005). Furthermore, changes in biodiversity raise concerns about the effects on ecosystem functioning and the provisioning of ecosystem services essential for human well‐being (Cardinale et al., 2012; Hooper et al., 2005; MEA, 2005).

Our understanding of biodiversity change at community and ecosystem level has improved by including functional characteristics or traits as measures of diversity (Diaz & Cabido, 2001). A *trait* is defined as any morphological, physiological, behavioural or life‐history characteristic affecting individual fitness and performance and can be either continuous (e.g., body size) or discrete (e.g., epifaunal‐ or infaunal‐living habit) (Diaz & Cabido, 2001; Laliberté & Legendre, 2010; Violle et al., 2007). The shift towards trait‐based approaches has generated quantitative measures that integrate multiple traits into single continuous indices, which allow for assessing multiple facets of functional change, such as richness, dominance (i.e., evenness) and dispersion (i.e., variation) of traits (Gagic et al., 2015; Laliberté & Legendre, 2010; Villéger, Mason, & Mouillot, 2008). However, to understand functional changes over time such indices are not enough, as they represent “snapshots in time” and do not encompass community dynamics (Collins et al., 2008; Hallett, Jones, Andrew, MacDonald, & Jones, 2016). For example, although the overall diversity within a community (α‐diversity) is not changing consistently through time, the rate of change in community composition (temporal β‐diversity, or so‐called turnover) might (Dornelas et al. 2014). Calculations of functional trait turnover, the change in trait composition between subsequent years, have mostly relied on measures applied to capture temporal species‐based presence/absence dissimilarities (Hewitt, Norkko, Kauppi, Villnäs, & Norkko, 2016; Villéger, Grenouillet, & Brosse, 2013). But the temporal change that a community experience is a result of changes in the abundance or biomass of each species in the community (Schimadzu et al. 2016). Recent developments for quantifying temporal taxonomic community turnover could also be fruitful for progressing assessments of functional trait turnover, as they encompass both identity and density on a community level (Hallett et al., 2016, Hillebrand et al., 2018, Schimadzu et al. 2015).

Our ability to mechanistically comprehend and predict functional changes and dynamics in real‐world ecosystems, despite recognizing the significance of them (Hillebrand & Matthiessen, 2009), are still impeded by several aspects. First, the focus on single trophic levels or specific organism groups restricts us from scaling up to encompass entire food webs and generalizing across ecosystems (Reiss, Birdle, Montoy, & Woodward, 2009; Thebault & Loreau, 2003). Only few studies have quantified temporal changes in both marine prey and consumer diversity (Katano, Doi, Eriksson, & Hillebrand, 2015), and fewer still in natural marine ecosystems (Englund, Rydberg, & Leonardsson, 2008; Nordström, Aarnio, Törnroos, & Bonsdorff, 2015; Olsson, Bergström, & Gårdmark, 2013). Second, adequately long time series, spanning several decades, are required to observe community and ecosystem change but are unfortunately often unavailable (Koslow & Couture, 2015). Thus, our understanding of long‐term functional community change is far from complete. Here, we address this knowledge gap, with the aim of assessing long‐term functional (trait) changes and potential couplings between two key interacting trophic groups in the marine food web, namely fish and zoobenthos. We use unique long‐term quantitative data sets of fish and zoobenthos from three different coastal areas in the Baltic Sea (HELCOM, 2017), one of the world's most heavily exploited Large Marine Ecosystems (Sherman & Hempel, 2008) and Marine Ecoregions (Spalding et al. 2007). This ecosystem is known to be spatially and temporally variable, with a decrease in taxonomic and functional richness following a salinity gradient (Griffiths et al., 2017; Ojaveer et al., 2010; Törnroos et al., 2015). It thereby provides an ideal model system for assessing long‐term trends in functional diversity and dynamics within organism and trophic groups such as fish and zoobenthos. Given the predator–prey relationship and that the groups respond to similar local environmental drivers (Olsson, Bergström, & Gårdmark, 2012; Olsson et al., 2013), we explore the hypothesis that the functional community developments of fish and zoobenthos are interlinked, or related to each other, with some time lag. Thus, we expect a relationship between the trends in diversity (species richness, trait richness, evenness and dispersion) and dynamics (trait turnover and multi‐trait composition) of the two groups. More specifically we ask (a) *Are there long‐term changes in functional diversity and dynamics of marine fish and zoobenthos communities?* and (b) *Do functional changes in zoobenthos communities correspond to functional changes in the fish community?*

## 2 MATERIALS AND METHODS

### 2.1 Community data

To illustrate the pronounced, natural gradient in biodiversity, and environmental characteristics of the Baltic Sea from south to north (Griffiths et al., 2017; SuppInfo A: Table S1, Table S2), three coastal areas: Vendelsö (hereafter “Kattegat,” 57°13′N; 12°04′E), Kvädofjärden (hereafter “Baltic Proper” 58°01′N;16°46′E) and Forsmark (hereafter “Bothnian Sea,” 60°26′N; 18°09′E), with comparable long‐term data series were chosen for the analysis (Olsson et al., 2012, 2013). Although we hereafter refer to the common and larger‐scale geographical names for the areas (Kattegat, Baltic Proper and Bothnian Sea), the data are primarily representative for the coastal parts of the basins. All three areas are used as monitoring reference areas by the Swedish coastal monitoring programmes, meaning, for example bottom trawling has not been allowed and other human pressures have been kept to a minimum (Bryhn, Franzén, Jonsson, & Lingman, 2017; HELCOM, 1996; Sundqvist, Svanfeldt, & Svensson, 2018). The fish and zoobenthos sampling sites are located in close proximity of each other (<5 km), and thus trophic interactions between fish and benthos are possible in each area (SuppInfo A: Table S1, Table S2).

Fish data (average catch in number of individuals per unit effort [net^‐1^ night^‐1^], CPUE, per species per year), collected by the Department of Aquatic Resources at the Swedish University of Agricultural Sciences (SLU), covered the time period 1976–2013 for Kattegat, 1971–2013 for the Baltic Proper, and 1975–2013 for the Bothnian Sea, with just three years of missing data overall (SuppInfo A: Table S1). Zoobenthic data (average number of individuals m^‐2 ^per taxa per year of soft‐bottom macrofauna) for the Bothnian Sea and the Baltic Proper were sampled by SLU, while data for Kattegat was acquired from the Swedish Meteorological and Hydrological Institute (SMHI). Zoobenthic data covered the time period 1972–2013 for Kattegat, 1980–2013 for the Baltic Proper, and 1976–2013 for the Bothnian Sea, with in total only five sampling‐years missing (SuppInfo A: Table S1). Both fish and zoobenthos taxa with a frequency of occurrence <5%, that is occurring only once or twice, and not in consecutive years, over the entire 40‐year time period, were excluded from the analyses as they were not considered as an established species in the area. In addition, for zoobenthos, all taxa contributing to in total at least 96% of the abundance per area were included. As the sampling gear do not catch all species in the area, the term “community” used refers to the sampled part of the community for both fish and zoobenthos. All data were ln‐transformed to improve statistical normality.

### 2.2 Trait information

Six overall traits were used for fish and zoobenthos, respectively, to objectively assess changes in functional diversity and trait dynamics over time (SuppInfo A: Table S3). These traits represent aspects related to feeding (e.g., diet, feeding mode and size), reproduction (e.g., reproductive frequency and type of development and egg type), population turnover (lifespan) and habitat affinity (environmental position). These traits summarize the ecological niche of the species and provide insight into the processes governing shifts in community structure. Trait information for fish was obtained from Fishbase (Froese & Pauly, [Link]) and Pecuchet, Törnroos, and Lindegren (2016). Zoobenthic trait information was obtained from Törnroos et al. (2015) and MARLIN (http://www.marlin.ac.uk/biotic/), supplemented with peer‐reviewed sources (collected in Garcia, 2010), and to a minor extent by expert judgement (A. Törnroos, L. Pecuchet personal communication). To harmonize across different trait types, as well as level of trait knowledge across fish and zoobenthos, we used traits in a discrete way (i.e. divided traits into categories/modalities). For traits with multiple categories, such as diet, we adopted the fuzzy coding approach (Chevenet, Doledec, & Chessel, 1994), which meant that an affinity of a species to a trait was one if the species express only one category, or a fraction of one if expressing several categories (Törnroos & Bonsdorff, 2012).

### 2.3 Calculating richness and diversity

We calculated the traditional species (taxon) richness (SRic) and three complementary multi‐trait functional indices: trait richness (TRic), functional evenness (FEve) and functional dispersion (FDis; Laliberté, Legendre, & Shipley, 2014, Schleuter, Daufresne, Massol, & Argillier, 2010). Trait richness describes the total number of trait categories expressed in the community for each year, while functional evenness informs on the density distribution among traits, and functional dispersion on the trait variability, or the spread of species and their density in trait space (Laliberté & Legendre, 2010; Mouillot, Nicholas, Villéger, Mason, & Bellwood, 2013). Higher functional evenness in a community means that density is more evenly distributed between trait categories (less dominance of certain traits), while a higher functional dispersion indicates that the community encompass differing densities of trait‐wise dissimilar species (Laliberté & Legendre, 2010; Mouillot et al., 2013). Thus, the latter two account for density differences between taxa in the community (SuppInfo B: Figure S1). The reason for using all three measures is that in combination, the indices describe key complementary dimensions of functional (trait) diversity (Laliberté & Legendre, 2010; Laliberté et al., 2014; Mouillot et al., 2013; Schleuter et al., 2010). In addition to this, the indices differ in how rapidly they inform on a change or disturbance, with the density‐weighted indices revealing impacts faster than trait richness (Chapin et al., 2000; Mouillot et al., 2013). The reason for this is that the driver of community change is often not strong enough to filter out specific traits right away, but generate significant differences in density (Boersma et al., 2016; Valdivia et al., 2017). Since the traits used are discrete, all trait‐based distance matrices used to calculate functional evenness and dispersion were calculated using Gower distances with Podani’s extension (Podani & Schmera, 2006).

### 2.4 Determining functional turnover and compositional changes

In addition to the aggregated biodiversity measures, we determined functional community (trait) turnover (Fturn) and multi‐trait dynamics. These analyses were performed per group and area on community‐weighted mean (CWM) traits (Laliberté et al., 2014), which were obtained by combining traits scores with the density of individuals. To estimate community trait turnover, we relied on the approach by Hallett et al. (2016), but instead of using taxonomic data and calculating turnover between all points in the time series, we applied the approach on the CWM trait data sets and calculated Euclidean distance between subsequent years, across the time series. This allowed us to estimate turnover that incorporated changes both in trait identity and density distribution from one year to the next. To obtain a measure of overall trait compositional changes, we conducted Dynamic Factor Analysis (DFA) on each CWM trait dataset (Holmes, Ward, & Wills, 2018). DFA is a multivariate time series technique especially designed for finding common trends in a set of time series (Zuur, Tuck, & Bailey, 2003). The advantage of DFA compared to other dimension‐reduction techniques like Principal Component Analysis (PCA), that are often applied on ecological time series (Planque & Arneberg, 2017), is that the temporal aspect (autocorrelation, stationarity) is explicitly taken into consideration (Zuur et al., 2003). For each CWM dataset (SuppInfo B: Figure S2), we tested different models containing from one to three trends and three different variance‐covariance matrices. The best model was selected based on Akaike's information criterion (AIC), which proved to contain three common trends and an equal variance and covariance matrix. This approach allowed us to summarize multi‐trait compositional changes by capturing the most important temporal dynamics in the trends, here only the first (T1) and the second (T2) are shown for clarity. For each trend, the loadings were also visualized (SuppInfo B: Figure S3).

### 2.5 Quantifying and comparing long‐term patterns

To quantify long‐term changes in diversity, we modelled the overall temporal trend in richness, the aggregate functional diversity indices, and the multi‐trait compositional changes (turnover, scores along T1 and T2, and individual CWM trait values). We fitted linear regressions on each measure, for each group and area separately, with year as a single predictor using either linear (Oksanen et al., 2015) or generalized least squares with a correlation structure (Gls, AR1; Pinheiro & Bates, 2000) that accounts for temporal autocorrelation (Pinheiro et al. 2018). The best model (including or excluding autocorrelation) was selected based on the AIC criterion (Bartón, 2018). In order to compare temporal dynamics of functional measures of fish and zoobenthos, we performed Pearson's paired‐sample correlation analysis between the two trophic groups for each measure and area. To account for potential lagged responses between prey and predators, correlations between the measures of fish and of benthos was tested both with and without 1‐year time lag for each group. However, since lagging did not improve the correlations, all results presented are nonlagged.

As a complement to the linear regression analysis and the correlation between groups, we also tested for potential nonlinear (step‐wise) shifts in the trophic groups and assessed timing of statistically relevant changes in each measure using change‐point analysis (Killick and Eckley 2014). We determine change‐points in time, related to shifted mean and variance in the time series using the binary segmentation method with a Bayesian information criterion (BIC) as penalty criteria (Killick and Eckley 2014). We identified both the gross change‐point of a time series (i.e. the one point in time where the index shift to a higher or a lower state, possible number of change‐points *Q* restricted to one) and the detailed variability, that is maximum number of points identified. Missing values were replaced by the average of the neighbouring two years. Assessing development in this way also allowed for comparison between groups and a reference to previously identified significant time periods of change in taxonomically‐based measures (Olsson et al., 2012, 2013).

All analyses described are conducted in the R environment (R Core Team, 2017).

## 3 RESULTS

### 3.1 Long‐term trends in richness and diversity

**Figure 1:**
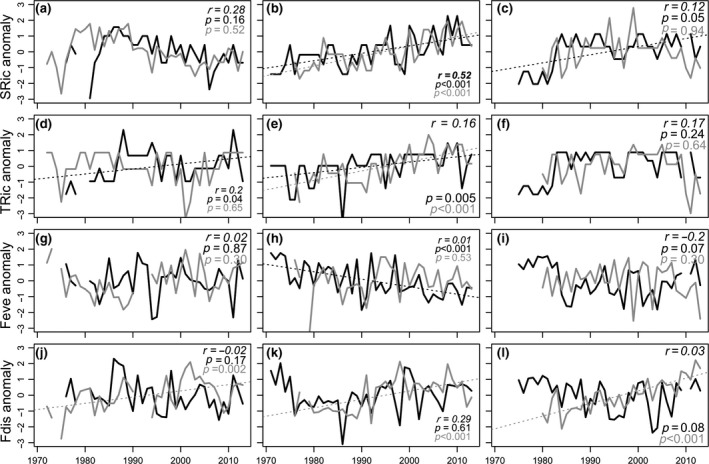
Long‐term trends in taxonomic and functional diversity indices. Species (a–c) and trait richness (d–f), functional evenness (g–i) and functional dispersion (j–l) of fish (black) and zoobenthos (grey) in Kattegat (left), Baltic Proper (middle) and the Bothnian Sea (right). To facilitate a comparison between fish and benthos the time series are shown as anomalies (zero mean and unit variance). Correlation coefficients between indices for fish and zoobenthos as well as p‐values for linear trends are found in the top (or bottom) right corner of each panel. Significant correlations are indicated in bold and significant trends with dashed lines

Over the roughly 40 years, fish species richness increased significantly in the Baltic Proper (*p* < 0.001) and Bothnian Sea (*p* = 0.05), while no linear change was found in Kattegat (Figure 1a–c). Species richness of zoobenthos only increased significantly in the Baltic Proper (Figure 1b). Trait richness of fish increased over time both in the Kattegat and in the Baltic Proper, while trait richness of zoobenthos increased only in the Baltic Proper (Figure 1d–f).

**Figure 2:**
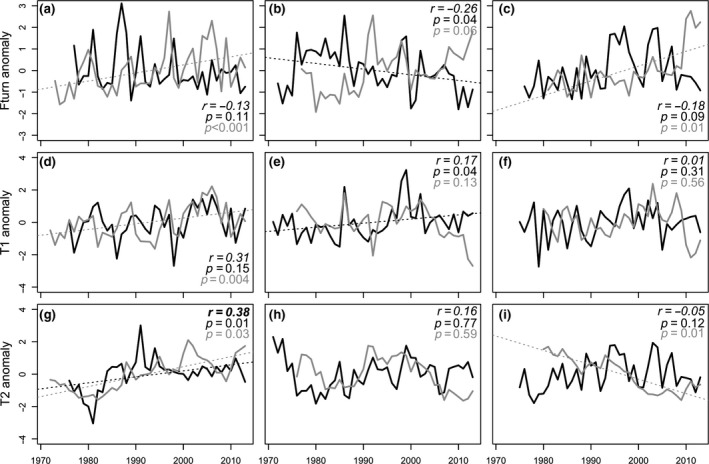
Changes in turnover and functional composition over time. Functional community turnover (a–c) and temporal trends based on DFA on community‐weighted trait values (CWM), T1 (d–f) and T2 (g–i), for fish (black) and zoobenthos (grey) in Kattegat (left), the Baltic Proper (middle) and the Bothnian Sea (right), respectively. Correlation coefficients between indices for fish and zoobenthos as well as *p*‐values for linear trends are found in the top (or bottom) right corner of each panel. Significant correlations are indicated in bold and significant trends with dashed lines

The density‐weighted trait evenness changed significantly (*p* < 0.001) in the Baltic Proper fish community, which became more uneven over time (Figure 2g–i). Changes in functional dispersion were only observed for zoobenthos, where an increase (*p* < 0.001) was found in all areas, hence, indicating a change over time of the abundant zoobenthic taxa in trait space (Figure 1j,k).

The change‐point analysis identified changes for fish prior to and in the early 1980s in all areas and diversity indices, except for functional dispersion in the Baltic Proper, for which a shift in 2007 was identified (SuppInfo A: Table S4). The changes in the zoobenthic communities were more variable between indices, and often displayed a spatial gradient in timing of changes, from earlier in Kattegat (late 1970s and early/mid‐1990s), to later in the Baltic Proper (early and late 2000s) and the Bothnian Sea (late 1990s and 2000s) (SuppInfo A: Table S4).

### 3.2 Temporal dynamics: functional turnover and compositional changes

Turnover of traits decreased significantly in the Baltic Proper fish community (*p* < 0.05), while an increase in turnover was found in both the Kattegat (*p* < 0.001) and Bothnian Sea (*p* < 0.01) zoobenthic communities (Figure 2a–c). The rate of change (intercept) in overall turnover was fastest in the northernmost area, the Bothnian Sea (Figure 2c).

Significant linear changes in the multi‐trait composition (T1 and/or T2) occurred in all areas but were group specific (Figure 2d–i). For fish, an increasing overall compositional change occurred in Kattegat (*p* < 0.05) and the Baltic Proper (*p* < 0.05) (Figure 2g, e). In the Kattegat fish community, these changes were accompanied by trait‐specific increases in especially small size (10–20 cm) and a generalist feeding habit (SuppInfo A: Table S5). Trait compositional changes in zoobenthos increased in Kattegat (*p* < 0.05) and decreased in the Bothnian Sea (*p* < 0.01) (Figure 2g, i). The increasing trend in Kattegat featured, similarly to the change in the fish community, an increase in small body size (0–10 mm), a scavenging feeding and an epibenthic‐living habit (SuppInfo A: Table S5). The linear decrease in the Bothnian Sea zoobenthos was attributed to decreases in infaunal‐living and deposit‐feeding habits, but also increases in categories such as epibenthic‐living and predator‐feeding habits (SuppInfo A: Table S5).

Change‐point analysis of turnover corresponded with the results for the diversity indices in that shifts were found during the same time period within each group, particularly for the zoobenthic communities (SuppInfo A: Table S4). Compared to the patterns for diversity indices, change points in functional turnover occurred in the 1990s and 2000s, for both fish and zoobenthos (except in the Bothnian Sea), rather than in the 1970s and 1980s, as was found for the diversity indices (SuppInfo A: Table S4).

### 3.3 Trophic group interlinkages in community change

Species richness of fish and zoobenthos showed positively correlated temporal dynamics in two out of the three areas (Kattegat *r* = 0.28, Baltic Proper *r* = 0.52; Figure 1a, b). The functional measures, on the other hand, displayed no temporal correlation or indication of linkage in community change between the two trophic groups. For trait richness, there was low correlation between the two groups in all areas (Figure 1d–f). Neither were there any indication of linkage between the trophic groups in functional evenness and dispersion (Figure 1g–i). Functional community turnover showed contrasting trends between fish and zoobenthos, with no correlation in any of the areas (Figure 2a–c). On the other hand, change‐point analysis in Kattegat showed coinciding changes in turnover for both fish and zoobenthos during the first part of the 1990s, while there was no correspondence between the groups in the other two areas (SuppInfo A: Table S4). Different temporal dynamics of fish and zoobenthos were also evident in the multi‐trait compositional changes over time (Figure 2d–i, SuppInfo B: Figure S3). Only in Kattegat did the first two time trends of benthos and fish correlate (T1 *r* = 0.31, T2 *r* = 0.38).

## 4 DISCUSSION

### 4.1 Long‐term functional changes

**Figure 3:**
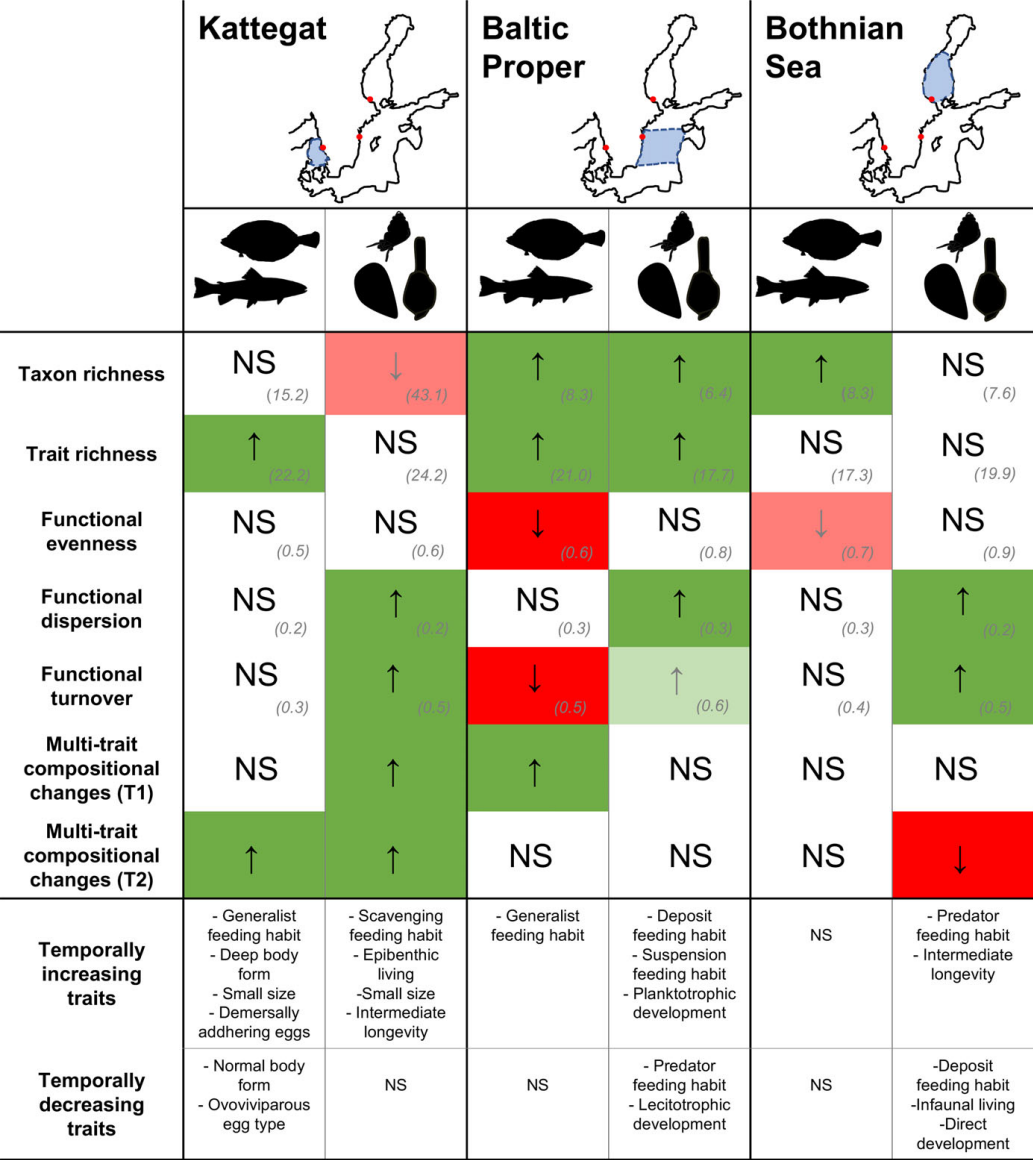
Summary of long‐term changes in species richness, functional diversity, turnover and multi‐trait composition across two trophic groups and three subsystems in the Baltic Sea. Maps show the three geographical areas with sampling area indicated in red. The data are primarily representative of the coastal parts of the basins. Upward arrows (↑, bright green) indicate a significant increase in a metric, while downward arrows (↓, bright red) indicate significant decrease. Trends (grey ↑↓, pale green or pale red), linearly increasing and decreasing traits, and nonsignificant changes (NS) are also given. Long‐term averages for species and trait richness, functional evenness and dispersion as well as functional community turnover are shown within lower‐case brackets [Colour figure can be viewed at http://wileyonlinelibrary.com]

By assessing long‐term trends in functional diversity, as well as trait turnover and multi‐trait composition, we identified gradual changes and distinct temporal dynamics between fish and zoobenthos communities at three coastal sites in the Baltic Sea (Figure 3). To our knowledge, this is the first study comparing long‐term trends and multi‐decadal dynamics in multiple dimensions of functional community change across trophic groups and areas. Previous trait‐based studies on long‐term functional community change have focused on single organism groups separately, either zoobenthos (Gogina, Darr, & Zettler, 2014; Neumann & Kröncke, 2011; Veríssimo et al., 2012; Weigel, Blenckner, & Bonsdorff, 2016) or fish (Baptista, Martinho, Nyjtrai, Pardal, & Dolbeth, 2015; Barcelo, Ciannelli, Olsen, Johannessen, & Knutsen, 2016; Dencker et al., 2017; Frelat et al., 2017), and particularly multi‐trait compositional changes on local scale (Clare, Robinson, & Frid, 2015; Frid & Caswell, 2015).

In order to adequately predict and mitigate such long‐term changes in biological assemblages, and ultimately associated ecosystem services, it is essential to understand not only how changes in species number, but also how shifts in density of organisms translates to potential functional changes in the system (Dornelas et al., 2013; Gagic et al., 2015; Hillebrand et al., 2018; Shimadzu, Dornelas, & Magurran, 2015). Our results highlight this by exemplifying and underlining the importance of comparative, temporal assessments of different complementary measures (e.g., taxonomic vs. functional, identity vs. density‐based) across trophic groups, for understanding biodiversity and functional change in a holistic way. More precisely, we need to know the mechanisms underpinning the functional changes, that is whether changes are driven by the richness and identity (key traits) and/or shifts in density of organisms (Hillebrand et al., 2018). These aspects have been investigated theoretically and experimentally for both terrestrial and aquatic systems (Cardinale et al., 2012; Morris et al., 2014), but rarely in comparison, empirically, over multi‐decadal time scales and across trophic levels, as we have done here. Our results specifically highlight three key aspects for understanding long‐term functional change and thereby the added value of functional trait‐based measures to traditional taxonomical ones. First, the results show that changes in species richness may be decoupled from changes in trait richness. For example, a significant increase in species richness but no significant change in trait richness was observed for the Bothnian Sea fish community but vice versa in the Kattegat (Figure 3). This contrasting pattern may indicate opposite changes in functional community composition in the two areas, potentially influencing their degree of functional redundancy (i.e. the number of functionally similar species sharing a similar set of traits; Walker, 1992) and resilience (i.e., their capacity to recover from change; Folke et al., 2004, Holling, 1973, Hillebrand, Bennett, & Cadotte, 2008). The latter is a common measure of stability or the potential to remain within an ecosystem state (McCann, 2000; Pimm, 1984). For the fish community in the Bothnian Sea, the increase in the number of functionally similar species may serve to increase functional redundancy and potentially also resilience as a community with more species is more likely to comprise species with traits that allow rapid recovery (Hillebrand et al., 2008; Lindegren, Checkley, Ohman, Koslow, & Goericke, 2016; Nyström, 2006). While for the Kattegat fish community, the increase in trait‐wise more dissimilar species may have led to a decrease in functional redundancy and potentially its resilience. As loss in resilience is difficult to detect until a community shifts to another state (Nyström, 2006), a change in the degree of resilience could potentially go unnoticed on a community level with few indications of even subtle long‐term functional trends in indices. Since the Kattegat ecosystem has been suggested to have undergone a regime shift, based on taxonomic single species assessments (Lindegren, Blenckner, & Stenseth, 2012), the next steps would be to specifically investigate the link between trait richness, redundancy and resilience for this system on a community level (Bouska, 2018; Carpenter, Walker, Anderies, & Abel, 2001; Lindegren et al., 2016). Secondly, our findings emphasize that changes in trait richness can be decoupled from changes in density‐weighted trait indices, in the same way as with taxonomic‐based indices (Hillebrand et al., 2018). This is important as density‐based indices are thought to respond more rapidly to changes than richness, and thus might inform on ecosystem function before species disappear completely (Chapin et al., 2000; Mouillot et al., 2013; Norberg, 2004). In our study, the change in dominance of traits in the community, rather than in the richness of expressed traits was more informative for understanding what underpinned the functional change. This was particularly evident in the zoobenthic community in Kattegat and Bothnian Sea, where significant long‐term changes in functional dispersion and multi‐trait measures, essentially portraying shifts in the degree and identity of dominant trait categories in trait space, were observed despite no significant change in trait richness (Figure 3). In turn, evenness, the direct complementary term to dominance, did significantly only change in the Baltic Proper fish community. As the number of traits (and also species in this case) increased over time, the frequency distribution of traits in the community became more uneven, meaning dominance increased, potentially increasing community resilience. Strong evidence from real‐world ecosystems for the conceptual link between evenness and resilience is lacking (Hillebrand et al., 2008), but experimental findings have linked decreases in evenness, that is increases in dominance, to community‐wide resilience in algal microcosm communities (Steiner, Long, Krumins, & Morin, 2006). Third, our results also show that an assessment of dynamics, that is trait turnover and multi‐trait compositional changes, in addition to the functional indices, is of importance for understanding the potential rate of change and type of functional change (identity of traits) that has occurred. Changes in trait composition have proven valuable for informing on long‐term changes in potential functioning in previous single trophic group studies, for example in North Sea zoobenthic infauna (Clare et al., 2015) and epifauna communities (Neumann & Kröncke, 2011) or coastal fish assemblages (Barcelo et al., 2016). In this study, long‐term changes in feeding habit and size where generally observed, suggesting potential shifts in the benthic and pelagic energy pathway (SuppInfo A: Table S5), specifically during certain time periods (SuppInfo B: Fig. S3). In particular, zoobenthic communities showed increases in either epibenthic predation and scavenging, or infaunal deposit‐ and suspension‐feeding, representing different trophic pathways (Figure 3). Thus, a combination of nonweighted and density‐weighted indices, as well as turnover is critical for detection and mechanistic understanding of complex spatiotemporal functional community changes. Disentangling such multiple dimensions of diversity and dynamics is therefore an important step in any type of ecological assessment (Hillebrand et al., 2018; Levin & Lubchenco, 2008).

### 4.2 Interlinkages across trophic groups

Individuals within the two trophic groups (fish and zoobenthos) have, in our case, had the potential to interact directly, through predatory–prey and/or other non‐trophic interactions through time, although this remains to be specifically assessed. A large portion of the fish community in the assembled data is benthivorous, or feeds on both zoobenthos and other fish species, especially in the Kattegat and Baltic Proper (SuppInfo A: Table S2). However, contrary to our hypothesis, we did not observe any strong relationship between the functional development of fish and zoobenthos communities, although similar trends were found for some biodiversity indices (Figure 3). This does not mean that trophic interactions do not influence functional community composition and the aspects of food‐web structure studied here, but that this was not identifiable using such broad community‐wide metrics. However, signs of functional changes related to food‐web interactions were observed in the measure of multi‐trait composition and individual trait changes. The similarity between fish and zoobenthos in individual traits showing long‐term changes, especially traits relating to size and feeding, suggests that the functional aspects of the cross‐trophic group linkages are worth investigating further. This applies especially in an ecosystem such as the Baltic Sea, showing large spatial variation in physicochemical and biological characteristics, as well as human impact (Griffiths et al., 2017; HELCOM, 2010, 2017). The key environmental drivers affecting community composition and food‐web dynamics in the Baltic Sea are salinity, temperature and oxygen (BACC II, 2015; Leppäranta & Myrberg, 2009; Lindegren, Andersen, Casini, & Neuenfeldt, 2014; Lindegren, Möllmann, Nielsen, & Stenseth, 2009; Pecuchet et al., 2016). These drivers have been shown to affect also the taxonomic composition of coastal communities in Kattegat (Olsson et al., 2012; Rosenberg, Loo, & Möller, 1995), Baltic Proper (Olsson et al., 2012, 2013) and Bothnian Sea (Kuosa et al., 2017; Olsson et al., 2012, 2013), but in different ways. Hence, these drivers are primary candidates for affecting the temporal patterns in the functional diversity indices of fish and zoobenthos presented here, and may also be reasons for the weak relationship between the trends in fish and zoobenthos found. A future study focused on the environmental linkage with particularly the density‐based functional indices and multi‐metric measures of community dynamics that showed significant long‐term changes is warranted.

This type of multi‐trophic assessment of different facets of temporal community change is also important for identifying, understanding and predicting larger ecosystem changes and dynamics, such as potential regime shifts that propagate across food web compartments (Folke et al., 2004, Spencer et al. 2011). In the Baltic Sea, studies on single species and taxonomic community composition have demonstrated abrupt changes in coastal areas (Olsson et al., 2012, 2013) or regime shifts in the offshore ecosystem (Blenckner et al., 2015; Casini et al., 2012; Kuosa et al., 2017; Lindegren et al., 2012; Möllman et al., 2009; Österblom et al., 2007), suggesting changes in the functioning of the system. In comparison, the long‐term gradual (step‐wise), linear trends and the low functional interlinkage we found in this study, provide no evidence for such abrupt changes in these coastal areas. This is in line with the study by Yletyinen et al. (2016) that found remarkable similarities of both coastal and offshore empirical food webs prior to and after the suggested Baltic Sea regime shift in 1980s. The coastal food webs maintained the dominant species interactions and showed no major shift at a community level despite changes in species composition (Yletyinen et al., 2016). These findings based on complexity theory and network modelling were suggested to be caused by high connectivity and absence of compartmentalization in the food webs, providing little support for system‐wide regime shifts. It is plausible that these are also the underlying mechanisms in the coastal communities studied here, reflected in maintenance of dominant traits (functional evenness) and/or trait variability (functional dispersion), especially in the zoobenthic prey community, which is then reflected in the gradual rather than abrupt changes in functional diversity and community dynamics (Figure 3). The link between long‐term changes in functional diversity, dynamics, and food web structure and abiotic drivers remains to be further explored, as these are all measures that provide an indication of the adaptive capacity, resilience and stability of the coastal assemblages and the ecosystem to future change.

To conclude, we have identified gradual long‐term trends in functional diversity (trait richness, evenness, dispersion), trait turnover, and overall multi‐trait community composition spanning a period of 40 years and two key trophic groups in three coastal marine areas. Although the linkage between fish and zoobenthic functional community change was weak, with timing of changes being area and trophic group specific, developments in specific fish and zoobenthos community traits, particularly size and feeding habits, suggest changes in trophic pathways. Apart from serving a baseline for functional change in the region and other coastal and estuarine ecosystems worldwide, the results highlight the need for multiple measures and cross‐trophic level assessments to understand empirical functional (trait) change. Thereby, our findings contribute to the general understanding of biodiversity change and can be useful for developing predictions and models of community change.
