# Supplementary material for: Four decades of functional community change reveals gradual trends and low interlinkage across trophic groups in a large marine ecosystem
Source: Glob Chang Biol. 2019 Feb 20;25(4):1235–46. doi: 10.1111/gcb.14552 (PMC6850384; doi:10.1111/gcb.14552)
Supplement: Supplementary file 1 [file GCB-25-1235-s001.pdf]

SUPPLEMENTARY INFORMATION B

Supplementary figures

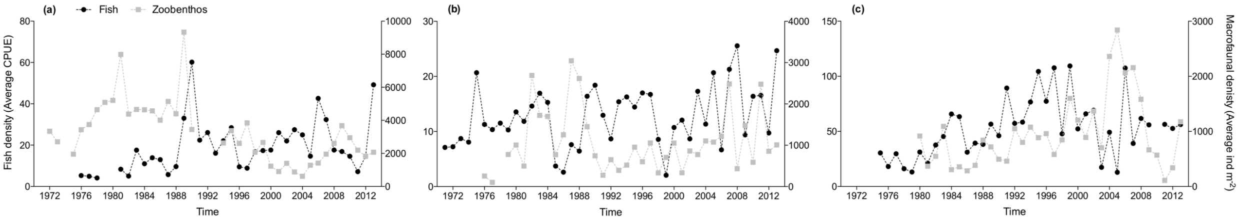

Figure S1. Density of fish and zoobenthos over time. Density trend of fish (average CPUE) and zoobenthos (Average abundance, m^{-2}) in (a) Kattegat, (b) Baltic Proper, (c) Bothnian Sea.

(a) Kattegat, fish community

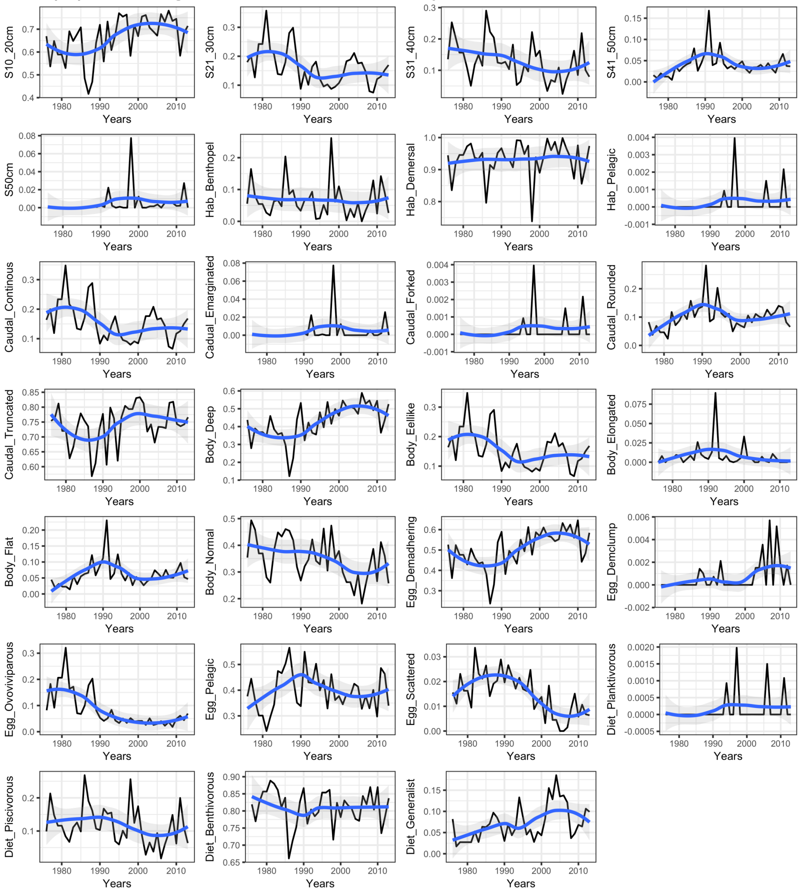

(b) Kattegat, zoobenthos community

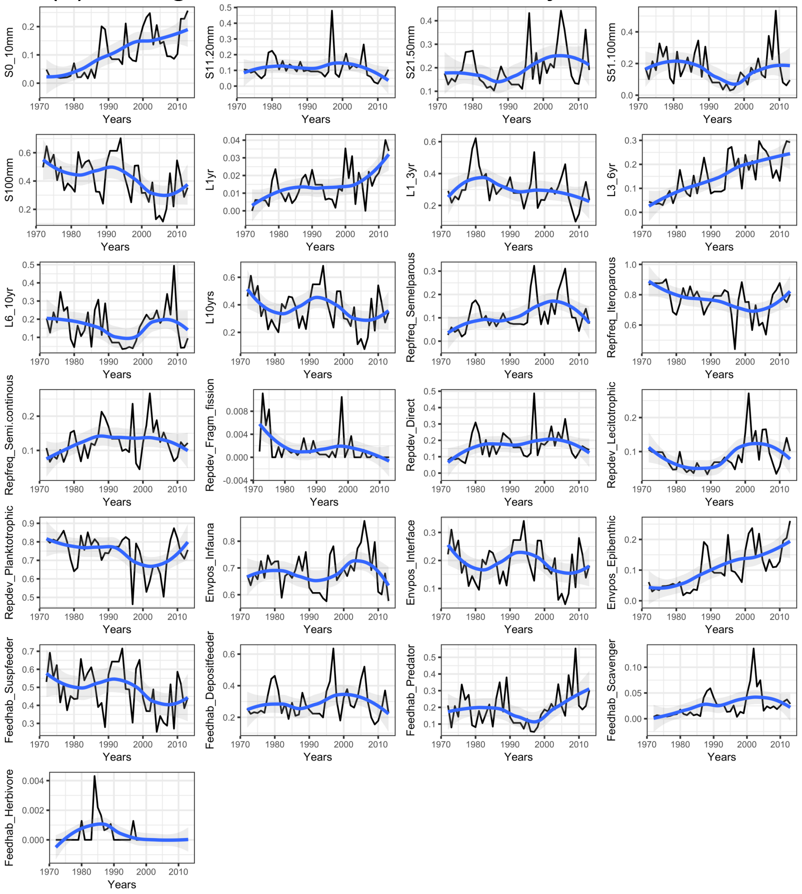

(c) Baltic Proper, fish community

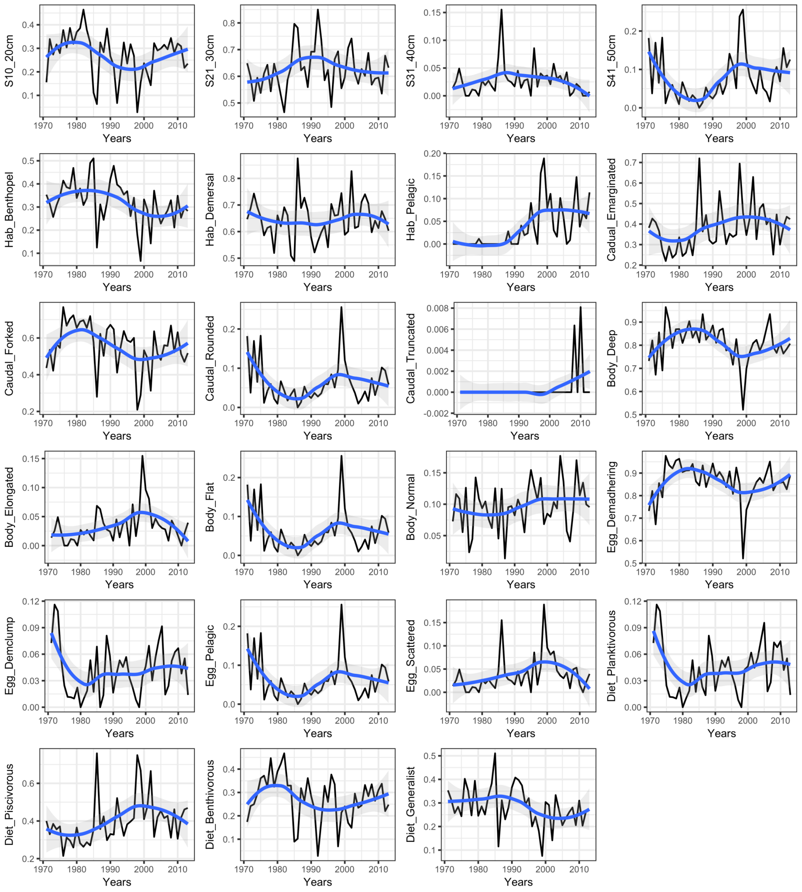

(d) Baltic Proper, zoobenthos community

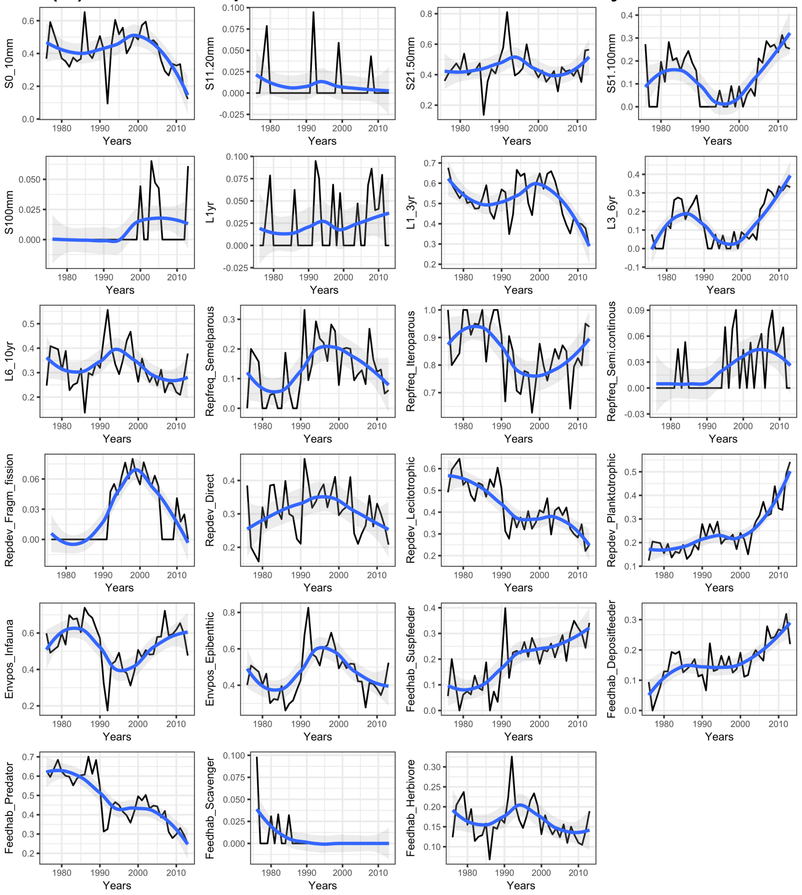

(e) Bothnian Sea, fish community

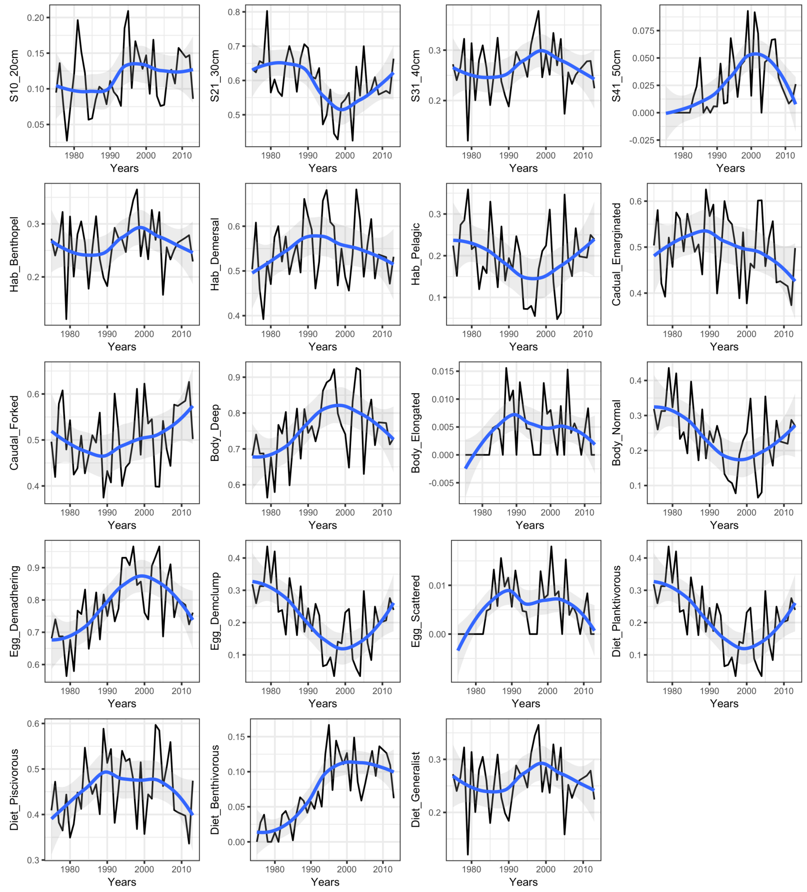

(f) Bothnian Sea, zoobenthos community

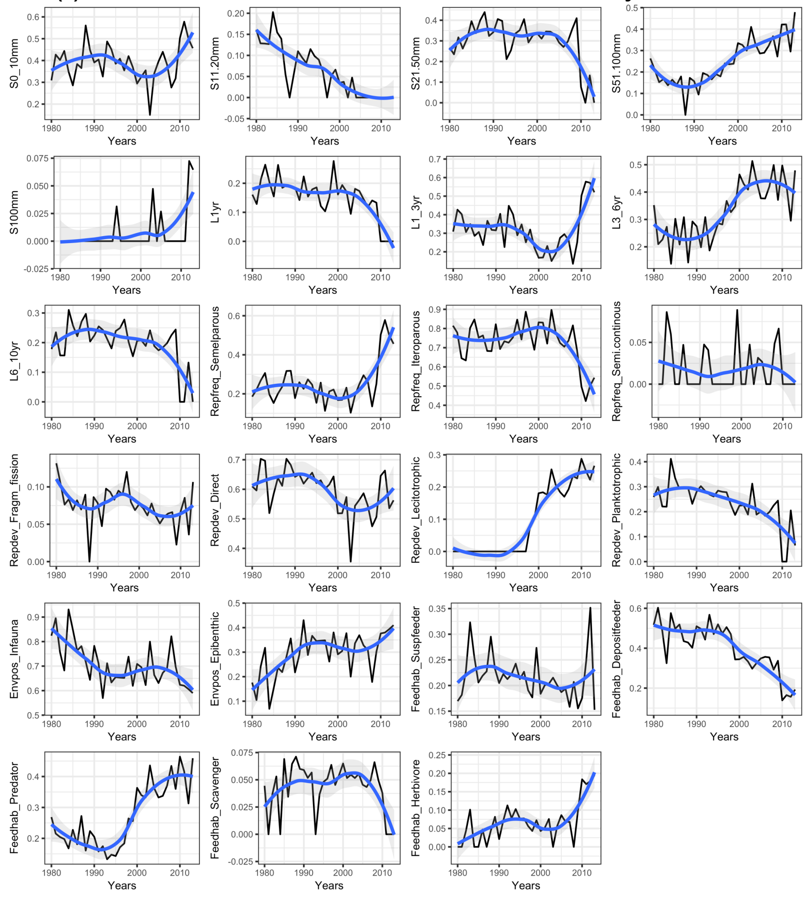

Figure S2. Temporal changes in individual trait categories for fish and zoobenthos in the three coastal areas. Temporal changes in individual trait categories (Community-weighted mean traits, CWMs) in Kattegat (a-b), Baltic Proper (c-d) and Bothnian Sea (e-f) for fish and zoobenthos, respectively. Trend lines (blue) and confidence intervals are produced with the loess method in R.

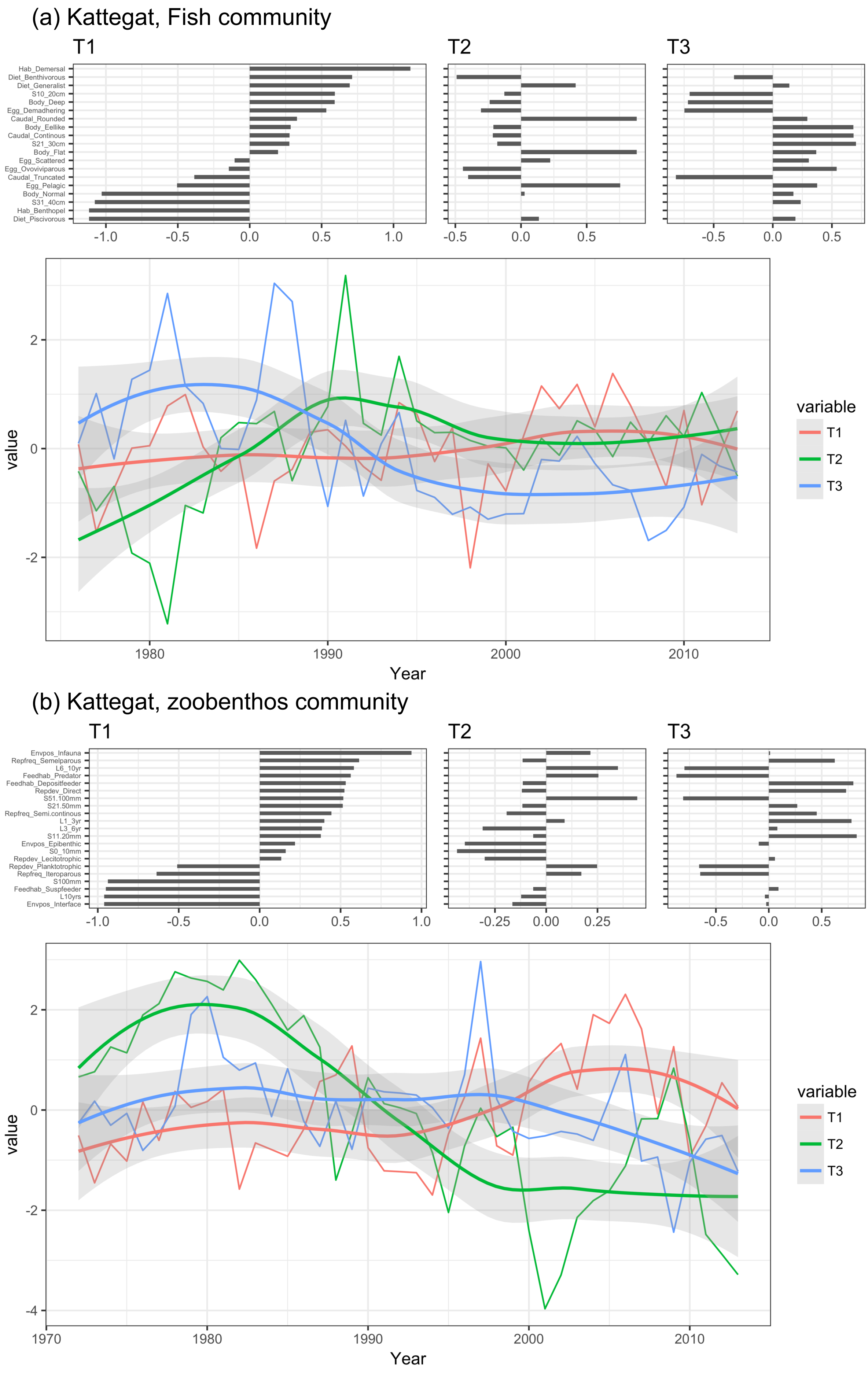

(a) Kattegat, Fish community
T1
T2
T3
Hab_Demersal
Diet_Benthivorous
Diet_Generalist
S10_20cm
Body_Deep
Egg_Demadhering
Caudal_Rounded
Body_Eellike
Caudal_Continous
S21_30cm
Body_Flat
Egg_Scattered
Egg_Ovoviviparous
Caudal_Truncated
Egg_Pelagic
Body_Normal
S31_40cm
Hab_Benthopel
Diet_Piscivorous
-1.0
-0.5
0.0
0.5
1.0
value
Year
1980
1990
2000
2010
2
0
-2
variable
T1
T2
T3
(b) Kattegat, zoobenthos community
T1
T2
T3
Envpos_Infauna
Repfreq_Semelparous
L6_10yr
Feedhab_Predator
Feedhab_Depositfeeder
Repdev_Direct
S51.100mm
S21.50mm
Repfreq_Semi.continous
L1_3yr
L3_6yr
S11.20mm
Envpos_Epibenthic
S0_10mm
Repdev_Lecitotrophic
Repdev_Planktotrophic
Repfreq_Iteroparous
S100mm
Feedhab_Suspfeeder
L10yrs
Envpos_Interface
-1.0
-0.5
0.0
0.5
1.0
-0.25
0.00
0.25
value
Year
1970
1980
1990
2000
2010
2
0
-2
-4
variable
T1
T2
T3

(c) Baltic Proper, fish community

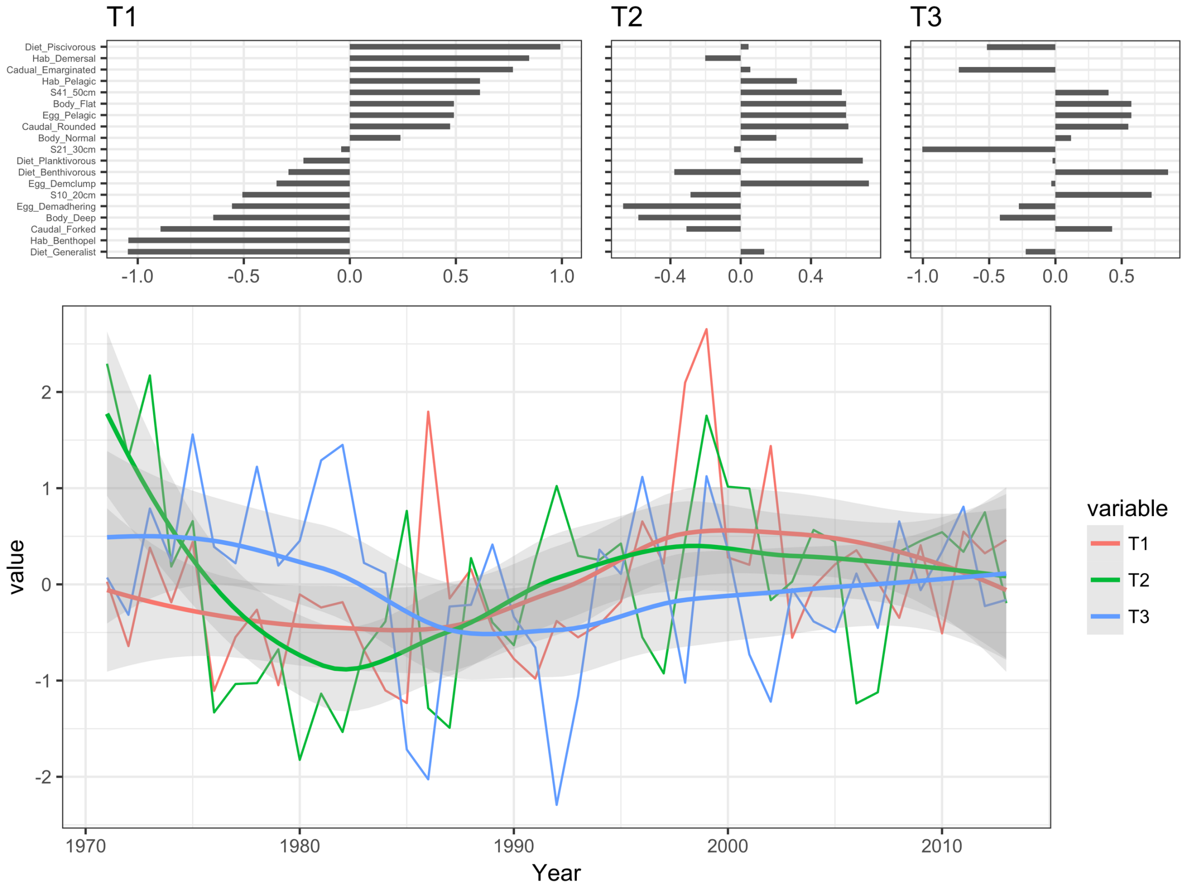

(d) Baltic Proper, zoobenthos community

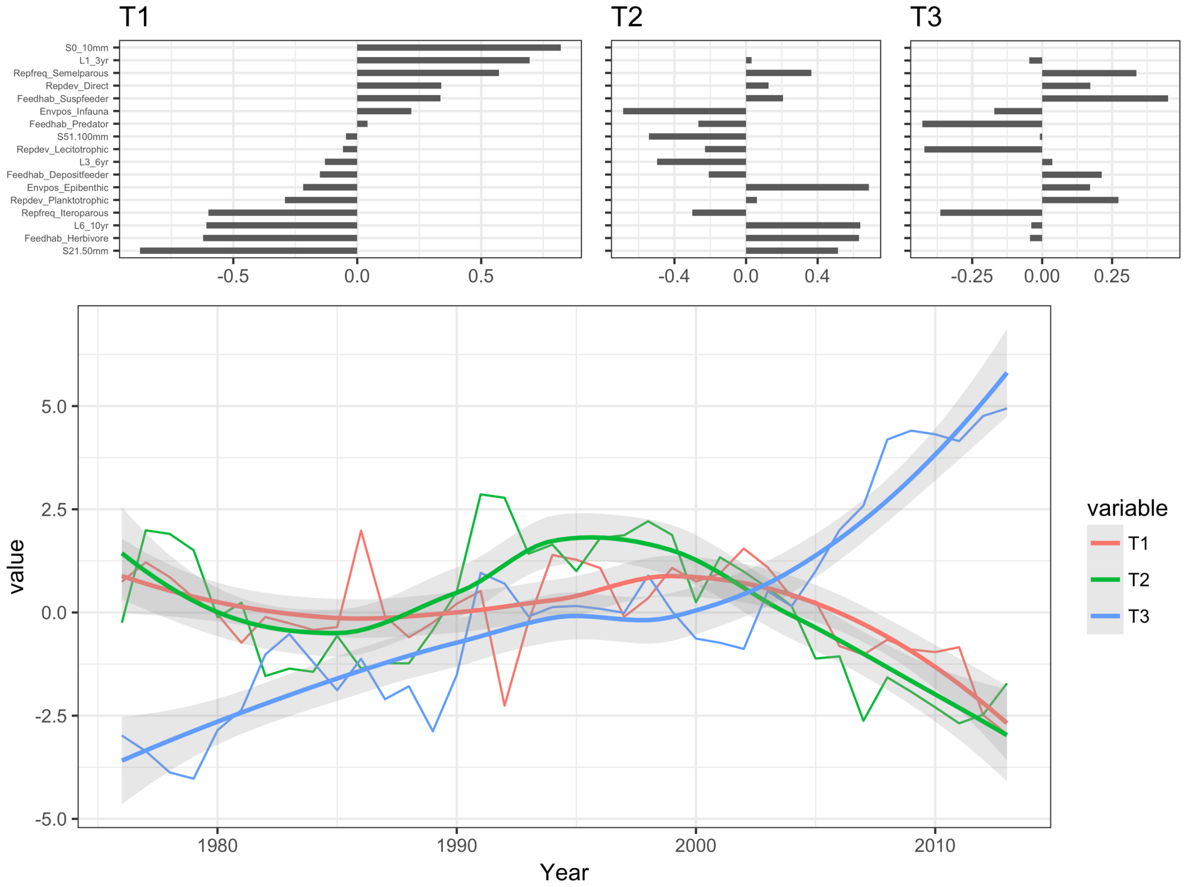

(e) Bothnian Sea, fish community

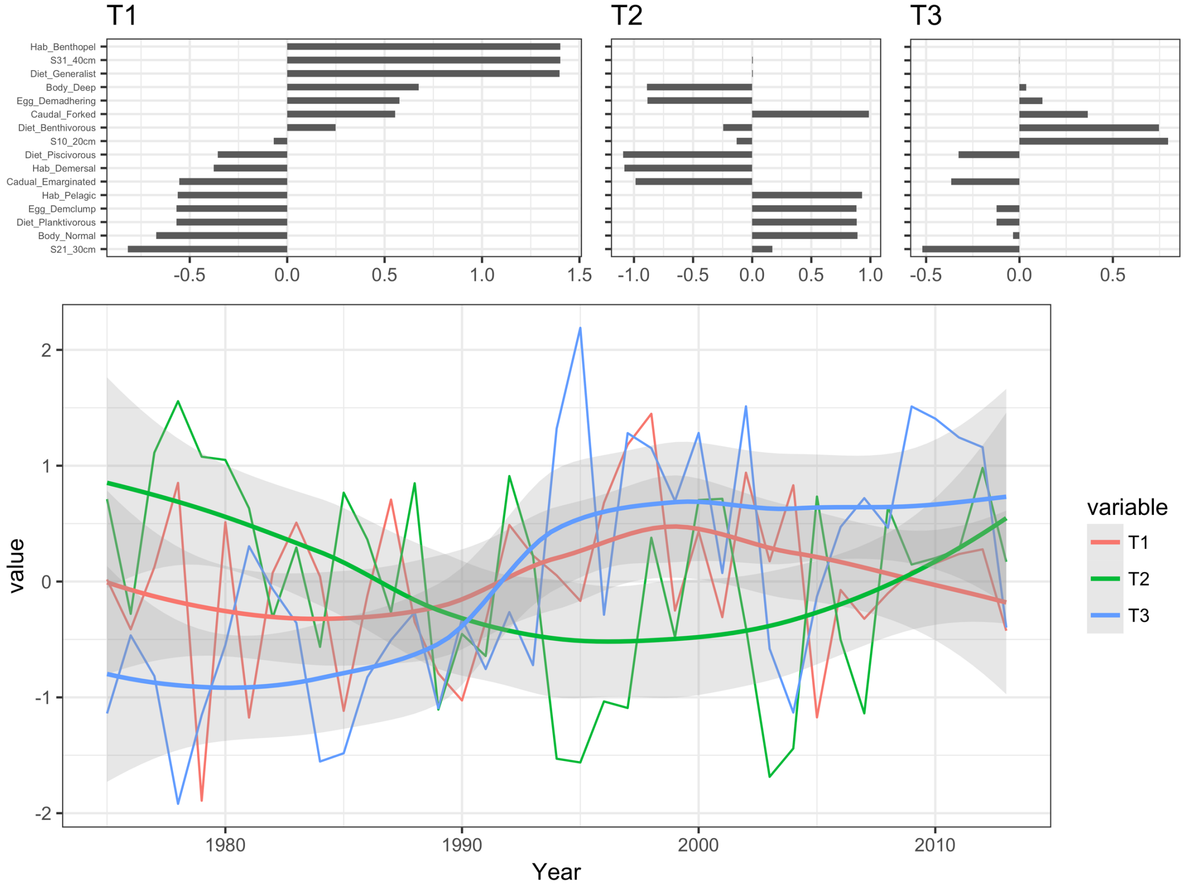

(f) Bothnian Sea, zoobenthos community

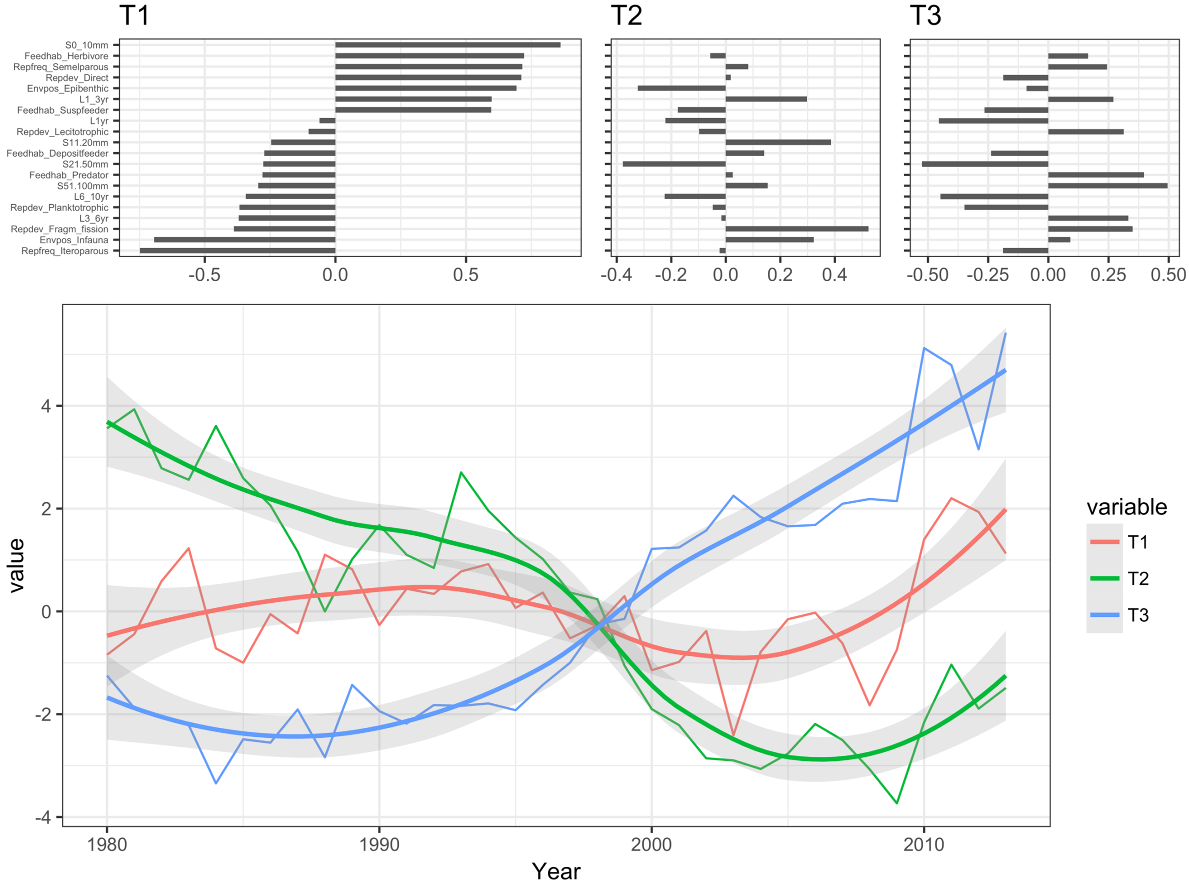

Figure S3. Multi-trait compositional changes of fish and zoobenthos. Time series trends (T1, T2, T3) and factor loadings (trait categories) in Kattegat (a-b), Baltic Proper (c-d) and Bothnian Sea (e-f) for fish and zoobenthos, respectively. Factor loading labels are arranged in decreasing order along time series trend T1. Trends and factor loadings are computed using DFA in the program R.
