# Supplementary material for: Four decades of functional community change reveals gradual trends and low interlinkage across trophic groups in a large marine ecosystem
Source: Glob Chang Biol. 2019 Feb 20;25(4):1235–46. doi: 10.1111/gcb.14552 (PMC6850384; doi:10.1111/gcb.14552)
Supplement: Supplementary file 2 [file GCB-25-1235-s002.pdf]

SUPPORTING INFORMATION (A)

Supplementary tables

Table S1. Information about fish and macrofaunal data included in the study. Data provider, sampling design (time period, sampling frequency, number of stations), gear, type of data and area characteristics.

Area	Trophic group	Data provider / Ref.	Timespan & month	Sample frequency & # stations	Depth	Sampling gear	Data type	Area description
**Kattegat** (Vendelsö)	Fish	SLU/ Olsson et al. 2012 ICES JMS	1976-2013 (missing data: 1979,1980) August	2 nets/sampling time, 9-12 nights/station/year, 6 fixed stations	2-5 m	Fykenet (fish down to 9 cm length)	Abundance (CPUE)	Shallow, open archipelago, max depth 10m, salinity: 17-20,
	Macrofauna	SMHI national data host /Mats Blomqvist – Hafok Ab	1972-2013, (missing data, 1991,1992,1993) March-June, December & November 1972 & 1976	3 replicate samples/ 1 station/year,	21 m	Smith-McIntyre, 1 mm mesh size	Abundance (Average of 3 replicates, ind m^{-2})	Open coast, Soft bottom: sand or mud, above halocline
**Baltic Proper** (Kvädöfjärden)	Fish	SLU/ Olsson et al. 2012 ICES JMS	1971-2013 August	18 nets/1 time/year, 4 fixed stations	15-20 m	Gillnets (7 diff. mesh sizes 21-60mm)	Abundance (CPUE)	Inner & outer archipelago part, 5-25 m depth, salinity: 6-8,
	Macrofauna	SLU/ Olsson et al. PLOS One 2013	1980-2013 (missing data:1978) May	5 samples within the area/year	22-24 m	Van Veen grab, 1 mm mesh size	Abundance (Average of 5 samples, ind m^{-2})	_____ " _____
**Bothnian Sea** (Forsmark)	Fish	SLU/ Olsson et al. 2012 ICES JMS	1975-2013 (missing: 2010) August	1 net/time, 3 nights/station, 3 fixed stations	2-5 m	Coastal survey nets (2 multimesh gillnets, 5 diff. mesh sizes 17-33mm)	Abundance (CPUE)	Shallow, 0-30 m, wave exposed, salinity: 3-4,
	Macrofauna	SLU/Olsson et al. PLOS One 2013	1976-2013 May	5 samples within the area/year	16 m	Van Veen grab, 1 mm mesh size	Abundance (Average of 5 samples, ind m^{-2})	_____ " _____

* Because sampling gear (fyke-, gill net series- or coastal survey nets) and design (number of nets, times and replicate stations per year) differed between areas (Table S1, for more detail see Olsson et al. 2012), we calculated an average per species and year across all stations to obtain a general estimate per area that was used in the analysis (Olsson et al. 2012).
* In Kattegat, three replicate samples were taken at the same station annually with a Smith-McIntyre grab sampler (1 mm mesh size) in March-June (except 1972 and -76 when samples were taken in December and November, respectively) at 21 meters (Table S1). In the Bothnian Sea and the Baltic Proper, five samples in the area were collected annually in May, with a Van Veen grab sampler (except for year 1980, -82, -83, when samples were obtained with an Ekman grab sampler that did not affect the results significantly) at 16 and 22-24 meters' depth, respectively, and sieved through a 1 mm mesh size.

References:

Ådjers K., Appelberg M., Eschbaum R., Lappalainen A., Minde A., Repecka R., Thoresson G. 1996. Trends in coastal fish stocks of the Baltic Sea. Boreal Environment Research 11: 13-25.

HELCOM 2008. Guidelines for HELCOM coastal fish monitoring sampling methods. http://www.helcom.fi/groups/monas/CombineManual/AnnexesC/en_GB/annex10/.

Thoresson, G. 1992. Handbok för kustundersökningar. Kustrapport: 1992: 4, in Swedish. https://www.fiskeriverket.se/download/18.2fd63c72114a6399bf68000585/PM029+handbok+recip.pdf

Thoresson, G. 1996. Guidelines for coastal fish monitoring. Swedish Board of Fisheries, Kustrapport 1996:2. https://www.fiskeriverket.se/download/18.2fd63c72114a6399bf68000641/PM087-eng+hand+ 1996-2.pdf

Supplementary Table S2. Total number of species (taxa), dominating species in terms of frequency of occurrence and density are presented per area and trophic group. Percentage of fish species expressing a benthivorous feeding habit in the three areas.

Area	Trophic group	Tot. # of taxa	Dominating taxa - Frequency (present >50% of yrs)	Dominating taxa – Density (contributing to >80% of density over yrs)	Percentage of fish with a benthivorous feeding habit
**Kattegat** (Vendelsö)	Fish	25	*Anguilla anguilla* (silver & yellow), *Ciliata mustela, Ctenolabrus rupestris, Gadus morhua, Gobius niger, Labrus bergylta, Merlangius merlangus, Platichthys flesus, Pleuronectes platessa, Solea solea, Symphodus melops, Taurulus bubalis, Myoxocephalus quadricornis, Zoarces viviparus* (tot: 14 taxa)	*Symphodus melops, Ctenolabrus rupestris* (tot: 2 taxa)	40% strictly benthivorous sp.<br><br>40% both piscivorous & benthivorous sp.
	Macrofauna	123	*Ampelisca brevicornis, Ampelisca tenuicornis, Ampharete baltica, Amphiura filiformis, Arctica islandica, Heteromastus filiformis, Chaetozone* sp., *Corbula gibba, Cylichna cylindracea,* Edwardsiidae, *Diplocirrus glaucus, Glycera alba, Goniada maculate, Hyala vitrea, Eudorella truncatula, Echinocardium cordatum, Rhodine gracilior, Mysella bidentate, Nephtys hombergii, Ophelina acuminate, Ophiura* sp., *Scoloplos armiger, Galathowenia oculata, Levinsenia gracilis, Pectinaria auricoma, Philine sp., Philine aperta, Phoronidae, Harpinia antennaria, Scalibregma inflatum, Abra nitida, Sphaerodorum flavum, Spiophanes bombyx, Thyasira flexuosa, Turritella communis* (tot: 36 taxa)	*Amphiura filiformis, Diplocirrus glaucus, Mysella bidentata, Nephtys hombergii, Scoloplos armiger, Galathowenia oculata, Pholoe inornata, Pholoe minuta,* Phoronidae, Prionospio fallax (tot: 10 taxa)	
**Baltic Proper** (Kvädöfjärden)	Fish	17	*Blicca bjoerkna, Esox lucius, Gymnocephalus cernuu, Leuciscus idus, Perca fluviatilis, Platichthys flesus, Rutilus rutilus* (tot: 7 taxa)	*Blicca bjoerkna, Perca fluviatilis, Rutilus rutilus* (tot: 3 taxa)	8 % strictly benthivorous sp.<br><br>20 % both piscivorous & benthivorous sp.
	Macrofauna	16	*Macoma balthica, Monoporeia affinis, Halicryptus spinulosus,* Chironomidae spp. (Tot: 4 taxa)	*Macoma balthica, Monoporeia affinis* (tot: 2 taxa)	
**Bothnian Sea** (Forsmark)	Fish	9	*Blicca bjoerkna, Clupea harengus membrans, Esox lucius, Gymnocephalus cernuu, Perca fluviatilis, Rutilus rutilus, Sander lucioperca* (tot: 7 taxa)	*Perca fluviatilis, Rutilus rutilus* (tot: 2 taxa)	4 % strictly benthivorous sp.<br><br>4 % both piscivorous & benthivorous sp.
	Macrofauna	15	Oligochaeta spp., *Saduria entomon, Monoporeia affinis, Corophium volutator, Potamopyrgus antipodarum, Macoma balthica* (tot: 6 taxa)	*Marenzelleria* spp., *Macoma balthica,* Oligochaeta spp. (tot: 3 taxa)	

Table S3. Traits and trait categories for fish and zoobenthos. Taxa are coded for trait categories based on fuzzy coding and affinity to a certain category of a trait is a fraction of 1.

Trophic group	Trait	Trait categories	Description /Relevance	Labels
**Fish**	Size	10-20cm	Mean length	S10_20cm
		21-30cm		S21_30cm
		31-40cm		S31_40cm
		41-50cm		S41_50cm
		>50 cm	Larger than 50 cm	S50cm
	Habitat	benthopelagic		Hab_Benthopel
		demersal		Hab_Demersal
		pelagic		Hab_Pelagic
	Feeding habit/ Diet	generalist	Feeds on detritus, plants, macrofauna	Diet_Generalist
		piscivorous		Diet_Piscivorous
		planktivorous		Diet_Planktivorous
		benthivorous		Diet_Benthivorous
	Egg type	demersal adhering eggs		Egg_Demadhering
		demersal mass clump		Egg_Demclump
		demersal scattered eggs		Egg_Scattered
		pelagic bouyant		Egg_Pelagic
		ovoviviparous		Egg_Ovoviviparous
	Caudal shape	emarginated		Caudal_Emarginated
		forked		Caudal_Forked
		rounded		Caudal_Rounded
		truncated		Caudal_Truncated
		continous		Caudal_Continous
	Body form	deep		Body_Deep
		elongated		Body_elongated
		eel-like		Body_Eellike
		normal		Body_Normal
		flat		Body_Flat
**Macrofauna**	Size	0-10mm		S0_10mm
		11-20mm		S11_20mm
		21-50mm		S21_50mm
		51-100mm		S51_100mm
		>100mm	Larger than 100 mm	S100mm
	Environmental position	infauna	From top to deep	Envpos_Infauna
		interface		Envpos_Interface
		epibenthic		Envpos_Epibenthic
		bentho-pelagic		Envpos_Benthopel
	Feeding habit	suspension feeder		Feedhab_Suspfeeder
		deposit feeder		Feedhab_Depositfeeder
		predator		Feedhab_Predator
		scavenger		Feedhab_Scavenger
		herbivore		Feedhab_Herbivore
		parasite		Feedhab_Parasite
	Reproductive	direct		Repdev_Direct

		lecitotrophic<br>planktotrophic<br>fragmentation/fission		Repdev_Lecitotrophic<br>Repdev_Planktotrophic<br>Reqdev_Fragm_fission
	Reproductive frequency	semelparous	Reproduces only once then dies	Repfreq_Semelparous
		iteroparous	Reproduces on several discrete times	Repfreq_Iteroparous
		semi-continuous	Reproduces over an extended period	Reqfreq_Semi-continous
	Longevity	<1yr	Less than one year	L1yr
		1-3yrs		L1_3yrs
		3-6yrs		L3_6yrs
		6-10yrs		L6_10yrs
		>10yrs	More than 10 years	L10yrs

Table S4. Timing of change in measures of diversity and community dynamics of fish and zoobenthos. Change points in species richness (SRic), trait richness (TRic), functional evenness (FEve), functional dispersion (FDis), functional trait turnover (Fturn) and multi-trait compositional trends (T1 and T2) were assessed for the two groups in three coastal areas of the Baltic Sea. The identified change points in a measure, i.e. a specific year of identified change, are based on the mean and variance of the measure, and presented with the gross shift per time series highlighted in bold. Missing values within time series are averaged from the two neighbouring years. Timespan of the time series of each group and area is given within brackets.

	**Kattegat**		**Baltic Proper**		**Bothnian Sea**	
	Fish (1976-2013)	Benthos (1972-2013)	Fish (1971-2013)	Benthos (1980-2013)	Fish (1975-2013)	Benthos (1976-2013)
SRic	**-**	1976,**1989**	**1973,** 1999, 2010	**2000**	**1982**	**-**
TRic	**1983**, 1986	1996, 1998, 2006, **2008**	**1974**, 1986	**1996**	**1982**,1985	1997, **2009**
Feve	**-**	**1974,** 2010	**1974**, 1997	**1979**	**1983**	**-**
FDis	**-**	**1977**,2010	**2007**	**1979,** 1990	**1983**, 2007	**1998,** 2008
Fturn	**1991**	**1995**	-	1977, **1990**	1991,**1993**, 2007	1998,**2009**
T1	**-**	**1999**	**1995**,2002	1993, **2005**, 2010	**-**	1999, **2009**
T2	1983,**1994**	1975,**1987**, 1999	**1973**,1987	1981,1984, 1990, 1999, 2004, **2006**	**-**	1985, 1996, **1998**

Table S5. Long-term trait-specific changes in fish and zoobenthos. Significant linear changes (slope, p-value and AIC-value) in trait categories in each trophic group and area. Trait categories are arranged in decreasing order based on AIC- value. Note: no significant changes were observed in the Baltic Sea fish community.

Area	Trophic group	Trait Label (trait & category	Slope	p-value	AIC
**Kattegat**	Fish	Body_Normal	-0,015	0,050	37,50
		Egg_Demadhering	0,018	0,008	38,88
		S10_20cm	0,018	0,013	45,14
		Body_Deep	0,023	0,011	49,11
		Egg_Ovovivioarous	-0,052	0,003	69,90
		Diet_Generalist	0,030	0,051	70,64
	Zoobenthos	Envpos_Epibenthic	0,047	<0,001	66,12
		L3_6yrs	0,050	<0,001	69,94
		S0-10mm	0,063	<0,001	83,67
		Feedhab_Scavenger	0,047	0,012	95,36
**Baltic Proper**	Fish	Diet_Generalist	-0,011	0,050	70,24
	Zoobenthos	Feedhab_Predator	-0,041	<0,001	19,90
		Repdev_Lecitotrophic	-0,033	<0,001	28,69
		Repdev_Planktotrophic	0,036	<0,001	30,09
		Feedinghab_Depositfeeder	0,056	0,007	133,0
		Feedinhab_Suspfeeder	0,085	0,001	158,3
**Bothnian Sea**	Zoobenthos	Repdev_Direct	-0,014	0,042	24,96
		Feedhab_Predator	0,039	0,029	28,84
		Feedhab_Depositfeeder	-0,045	<0,001	35,92
		L3_6yr	0,036	<0,001	43,84
		Envpos_Epibenthic	0,028	0,003	56,51
		Envpos_Infauna	-0,028	0,003	56,56
